# Revolutionizing Oncology: A Comprehensive Review of Digital Health Applications

**DOI:** 10.7759/cureus.59203

**Published:** 2024-04-28

**Authors:** Samidha Borkar, Swarupa Chakole, Roshan Prasad, Spandan Bansod

**Affiliations:** 1 Medicine, Jawaharlal Nehru Medical College, Datta Meghe Institute of Higher Education & Research, Wardha, IND; 2 Community Medicine, Jawaharlal Nehru Medical College, Datta Meghe Institute of Higher Education & Research, Wardha, IND; 3 Obstetrics and Gynecological Nursing, Srimati Radhikabai Meghe Memorial College of Nursing, Datta Meghe Institute of Higher Education and Research, Wardha, IND

**Keywords:** telemedicine in oncology, patient data privacy, ai in cancer treatment, precision oncology, cancer care technology, digital health in oncology

## Abstract

Digital health is poised to revolutionize the field of oncology, offering innovative solutions that enhance diagnostics, treatment, and patient care. This comprehensive review delves into the multifaceted landscape of digital health in oncology, encompassing its definition, significance, applications, benefits, challenges, ethical considerations, and future trends. Key findings highlight the potential for early detection, personalized treatment, enhanced care coordination, patient empowerment, accelerated research, and cost efficiency. Ethical concerns surrounding privacy, equitable access, and responsible data use are discussed. Looking ahead, the future of digital health in oncology is bright, driven by advancements in artificial intelligence, virtual and augmented reality, predictive analytics, global collaboration, and evolving regulations. This review underscores the need for collaboration among stakeholders and a patient-centered approach to harness the transformative power of digital health, promising a future where the burden of cancer is lessened through innovation and compassionate care.

## Introduction and background

Digital health, often referred to as "eHealth" or "healthtech," represents the intersection of healthcare and technology, and its significance in the field of oncology cannot be overstated. Digital health in oncology encompasses a broad spectrum of technologies, strategies, and innovations to improve cancer prevention, diagnosis, treatment, and management using digital solutions. These solutions include, but are not limited to, electronic health records (EHRs), mobile health apps, wearable devices, telemedicine platforms, and artificial intelligence (AI)-driven analytics [1].

Digital health offers the potential for early cancer detection by analyzing extensive patient datasets encompassing genetic markers, lifestyle factors, and imaging data. This capability holds the promise of identifying cancer at its earliest, most treatable stages, paving the way for more timely interventions and improved treatment outcomes [2]. Digital health technologies unlock the era of personalized medicine in oncology. By delving into genetic and molecular data, these tools facilitate the development of highly individualized treatment plans. Patients benefit from therapies tailored to their unique genetic profiles, maximizing the effectiveness of treatments while minimizing adverse effects [3].

Digital health contributes to remote monitoring, which is a boon for cancer treatment patients. Wearable devices and telemedicine enable continuous health tracking, reducing the necessity for frequent hospital visits. This enhances patient convenience and improves their overall quality of life, which is especially crucial for those with compromised health due to cancer [4]. Digital health generates vast volumes of data that fuel research and clinical trials. These invaluable datasets accelerate our comprehension of cancer biology, treatment effectiveness, and the development of novel therapies. With digital health as a catalyst, we stand on the brink of significant advancements in our fight against cancer, with the potential to transform outcomes for patients worldwide [5].

This review aims to delve into the evolving landscape of digital health in oncology, examining its current state, emerging trends, challenges, and potential for healthcare providers and patients. We will explore the various technologies and applications within digital health, their impact on oncology care, and the ethical and regulatory considerations associated with their implementation. Additionally, this review aims to shed light on the future directions of digital health in oncology and its transformative role in the fight against cancer.

## Review

Types of digital health applications in oncology

Diagnostics and Screening

Genomic sequencing: Genomic sequencing is a cornerstone of digital health applications in oncology. This powerful technique involves the comprehensive analysis of a patient's genetic makeup, providing valuable insights into genetic mutations and variations associated with cancer risk, prognosis, and treatment response. By deciphering the patient's unique genetic profile, oncologists can tailor treatment strategies to target specific genetic aberrations. Genomic sequencing is instrumental in identifying precision medicine approaches, allowing for the administration of targeted therapies that address the underlying genetic drivers of cancer. Furthermore, it aids in assessing cancer susceptibility, enabling early interventions and preventive measures for individuals at higher genetic risk. Overall, genomic sequencing represents a transformative tool in the fight against cancer, driving personalized treatment and improving patient outcomes [6].

Liquid biopsies: Liquid biopsies are minimally invasive diagnostic tests that analyze various biomarkers, including circulating tumor DNA and circulating tumor cells, present in blood or other bodily fluids. These tests have revolutionized cancer diagnostics by enabling early detection, monitoring treatment efficacy, and tracking disease progression. Liquid biopsies offer several advantages, including reduced invasiveness, faster results, and capturing tumor heterogeneity. Early cancer detection through liquid biopsies is pivotal, allowing for prompt interventions and potentially more effective treatments. Additionally, these tests provide a dynamic view of a patient's cancer status, facilitating real-time adjustments to treatment plans based on molecular changes within the tumor. Liquid biopsies represent a significant advancement in cancer screening and monitoring, offering hope for improved outcomes and quality of life for cancer patients [7].

Radiomics: Radiomics is a cutting-edge approach that harnesses advanced image analysis techniques to extract quantitative data from medical images, such as CT scans and MRIs. This data is then analyzed using machine learning algorithms, which can predict various tumor characteristics, treatment responses, and patient outcomes. Radiomics plays a crucial role in treatment planning and assessment, guiding oncologists to make informed decisions about therapies and interventions. By analyzing medical images comprehensively and quantitatively, radiomics assists in identifying specific tumor features, patterns, and changes over time. This information aids in tailoring treatment strategies to individual patients and monitoring treatment responses. Radiomics holds great promise for optimizing cancer care by providing valuable insights into tumor biology and response to therapy [8].

Treatment Decision Support

AI-driven clinical decision support systems: Digital health in oncology harnesses the power of AI-driven clinical decision support systems. These advanced systems integrate vast datasets, including patient data, medical literature, and clinical guidelines, to assist healthcare providers in making informed and personalized treatment decisions. AI-driven decision-support systems analyze this data to generate treatment recommendations tailored to each patient's unique profile. By considering factors such as genetics, disease stage, comorbidities, and treatment history, these systems help oncologists optimize therapy choices. They provide real-time insights, aiding healthcare providers in selecting the most effective treatments, predicting potential side effects, and identifying opportunities for intervention. AI-driven decision support enhances the precision and individualization of cancer care, ultimately improving patient outcomes and quality of life [9].

Drug discovery and development: Digital health technologies are increasingly pivotal in drug discovery and development, particularly in oncology. AI and computational modeling are leveraged to expedite the identification of potential drug candidates, predict drug interactions, and optimize clinical trial designs. These technologies accelerate the development of novel cancer therapies by streamlining the research and development process. AI algorithms analyze vast datasets, including genomic information and biological data, to identify potential therapeutic targets. This data-driven approach enhances the efficiency of drug discovery, reducing the time and cost required to bring new cancer treatments to market. Additionally, AI can predict drug interactions and adverse effects, aiding in the development of safer and more effective cancer drugs. Overall, digital health is revolutionizing the landscape of cancer treatment by facilitating the discovery and development of innovative therapies [10].

Remote Monitoring

Wearables and sensors: Digital health leverages wearable devices, such as smartwatches and fitness trackers equipped with various sensors, to continuously monitor vital signs, physical activity, and sleep patterns. In oncology, these wearables play a crucial role in tracking the health and well-being of cancer patients. Patients can wear these devices, allowing healthcare providers to monitor their vital signs remotely, detect potential complications, and track their response to treatment. For example, wearables can monitor heart rate, blood pressure, and oxygen levels, providing real-time data to healthcare teams. This continuous monitoring facilitates early intervention when irregularities are detected, leading to more personalized and timely care. It also empowers patients by giving them insights into their health status, fostering a sense of control and well-being during their cancer journey [11].

Telemedicine and virtual care: Telemedicine and virtual care platforms have emerged as essential components of digital health, providing cancer patients with remote access to healthcare services. These services encompass various offerings, including consultations with oncologists, follow-up appointments, and mental health support. Telemedicine and virtual care platforms facilitate secure and convenient communication between patients and healthcare providers, ensuring patients receive the care they need without needing in-person visits. This approach is particularly crucial for cancer patients, many of whom have compromised immune systems due to treatments like chemotherapy. Remote consultations and care allow patients to access timely medical advice and support while reducing their exposure to infectious risks in healthcare settings. Furthermore, these digital platforms enhance access to specialists, enabling patients in remote or underserved areas to receive specialized care without requiring extensive travel [12].

Patient Support and Engagement

Mobile apps and patient portals: Digital health in oncology has brought forth an array of mobile applications and patient portals designed to support and engage cancer patients throughout their journey. These tools offer patients valuable resources, including educational materials, medication reminders, symptom tracking, and seamless communication with their healthcare teams. Mobile apps empower patients by putting essential information and tools at their fingertips, enabling them to actively participate in their care. For instance, patients can access educational content to better understand their condition and treatment options, set medication reminders to stay on track with their therapies, and monitor symptoms to provide timely updates to their healthcare providers. These digital resources enhance patient engagement and empower individuals to take a more active role in managing their health during their cancer experience [13].

Supportive care services: Digital health extends its reach to encompass supportive care services for cancer patients. This includes access to online support groups, counseling services, and palliative care resources. These digital offerings address the holistic needs of cancer patients, recognizing that cancer care encompasses not only medical aspects but also emotional, social, and psychological well-being. Online support groups create a sense of community, connecting patients with others who share similar experiences and providing a platform for sharing advice and emotional support. Digital counseling services offer convenient access to mental health professionals, helping patients cope with the emotional challenges often accompanying a cancer diagnosis. Palliative care resources, accessible through digital health, guide managing pain and improving quality of life, offering comfort and support to patients and their families [14].

Data Management and Analytics

EHRs: EHRs play a pivotal role in digital health by centralizing patient health information in a digital format. EHRs are comprehensive repositories that house a patient's medical history, diagnoses, treatment plans, medications, test results, and more. In the context of oncology, EHRs provide oncologists and healthcare providers with quick and easy access to accurate, up-to-date patient data. This facilitates seamless communication among healthcare team members and ensures that all providers involved in a patient's care have a unified view of the patient's health journey. EHRs enhance care coordination by enabling healthcare providers to make data-driven decisions, reduce redundancy in testing, and streamline treatment planning. They also improve patient safety by reducing the risk of medical errors associated with paper-based records [15].

Big data and machine learning: The utilization of big data analytics and machine learning is transforming oncology by enabling the aggregation and analysis of vast datasets. These datasets encompass diverse patient information, including genetic data, treatment outcomes, imaging studies, etc. Through big data analytics and machine learning algorithms, researchers and healthcare providers can identify patterns, trends, and treatment insights that may have otherwise gone unnoticed. These technologies hold immense potential for improving cancer care and research. They can help identify risk factors for cancer, predict patient responses to treatments, optimize treatment plans, and even discover new therapeutic targets. Additionally, machine learning models can evolve and adapt by continuously learning from new data, providing clinicians with the latest insights into cancer biology and treatment strategies [16].

Benefits of digital health in oncology

Improved Diagnostics and Personalized Treatment

Early detection: Digital health tools, including genomic sequencing and liquid biopsies, have revolutionized cancer diagnostics by enabling early cancer detection. These technologies can detect cancer at its earliest stages, often before symptoms manifest. This early detection is critical because it increases the likelihood of successful treatment outcomes. By identifying cancer in its nascent stages, clinicians can implement treatment strategies when the disease is most susceptible to intervention, potentially leading to better survival rates and improved patient quality of life [17].

Personalized treatment: Digital health is pivotal in tailoring cancer treatment plans to individual patients. Digital health solutions enable healthcare providers to create personalized treatment regimens by analyzing patients' genetic profiles and tumor characteristics. This approach considers each patient's unique genetic makeup and their cancer's specific characteristics. As a result, treatments can be optimized to target the underlying mechanisms driving the disease, maximizing therapeutic efficacy while minimizing side effects. Personalized treatment plans improve patient outcomes and enhance patient experience by reducing unnecessary treatments and their associated adverse effects [18].

Predictive analytics: Digital health leverages the power of machine learning and AI algorithms to predict treatment responses and guide real-time adjustments. These predictive analytics models analyze vast datasets, including patient profiles, genetic information, treatment histories, and outcomes. By continuously monitoring patient responses and adjusting treatment protocols based on data-driven insights, healthcare providers can optimize therapy. This approach ensures patients receive the most effective treatments while minimizing unnecessary interventions, reducing side effects, and conserving healthcare resources. Predictive analytics offer a dynamic and patient-centric approach to cancer treatment, enhancing its effectiveness and efficiency [19].

Enhanced Care Coordination

Integrated information: EHRs are a linchpin for enhancing care coordination within digital health. These electronic records centralize patient data, aggregating a comprehensive view of the patient's medical history, including diagnoses, treatments, medications, and test results. Importantly, this information is readily accessible to all healthcare providers involved in a patient's care, whether primary care physicians, oncologists, radiologists, or specialists. This integration streamlines communication and collaboration among healthcare teams, reducing the risk of errors and ensuring that everyone is working with the most up-to-date information. EHRs enable healthcare providers to make more informed decisions, tailor treatment plans to individual patient needs, and avoid redundant or conflicting interventions, ultimately leading to better-coordinated and safer care [20].

Remote consultations: Telemedicine and virtual care have emerged as indispensable tools for enhancing care coordination in oncology. These technologies enable oncologists and specialists to collaborate and consult remotely, bridging geographical distances and ensuring patients receive timely and coordinated care, regardless of location. Remote consultations allow healthcare teams to discuss cases, share expertise, and make joint decisions about treatment plans. This means faster access to specialized care and reduced diagnosis or treatment initiation delays for cancer patients. Moreover, it promotes continuity of care, allowing patients to remain connected to their trusted healthcare providers even if they need to seek expertise from a distant specialist. This flexibility in care delivery enhances the patient experience and improves overall care coordination within the oncology ecosystem [21].

Patient Empowerment and Engagement

Education and support: Digital health tools, including mobile apps, patient portals, and online resources, are potent enablers of patient empowerment and engagement in cancer care. These tools give cancer patients unprecedented access to information about their condition, treatment options, and self-care strategies. Through user-friendly interfaces, patients can learn about their diagnosis, understand the benefits and risks of various treatment approaches, and access a wealth of supportive resources, such as educational materials, forums, and peer support groups. This increased knowledge equips patients with the information they need to actively participate in their care decisions, ask informed questions, and collaborate more effectively with their healthcare teams. Patient empowerment through digital education fosters a sense of autonomy and confidence, improving overall patient experiences and outcomes [22].

Self-monitoring: Wearable devices and remote monitoring solutions are instrumental in promoting patient empowerment and engagement. These technologies empower cancer patients to take an active role in monitoring their health. In real-time, wearable devices can track vital signs, physical activity, and other health metrics. This real-time data lets patients understand their health status and recognize potential issues early. For example, patients can monitor their heart rate, sleep patterns, or physical activity levels to track recovery progress, identify signs of treatment-related side effects, or spot changes that may require medical attention. By enabling patients to play a more proactive role in managing their health, these digital tools contribute to a sense of control and self-efficacy, ultimately improving cancer patients' overall quality of life [23].

Research and Clinical Trials Advancements

Data-driven research: Digital health in oncology generates vast amounts of real-world patient data, offering a treasure trove for researchers. This data, encompassing diverse patient profiles, treatment outcomes, genetic information, and more, provides invaluable insights into cancer biology, treatment effectiveness, and side effects. Researchers can tap into this rich resource to accelerate the development of new cancer therapies and treatment strategies. By leveraging digital tools and analytics, they can identify patterns and correlations that may have remained hidden. This data-driven research approach enables scientists to make more informed decisions about treatment options, tailor therapies to individual patients, and discover innovative approaches to combat cancer. The result is a faster-paced and more targeted approach to cancer research, potentially bringing new treatments to patients more quickly [24].

Efficient clinical trials: Digital health technologies, particularly AI algorithms, play a pivotal role in advancing the efficiency of clinical trials in oncology. Identifying eligible patients for clinical trials is a critical yet often time-consuming process. AI-driven algorithms excel at sifting through large datasets to pinpoint individuals who meet the specific criteria for a trial. This expedites recruitment, reducing the time and resources required to enroll participants. Moreover, AI can assist in matching patients with the most suitable clinical trials, enhancing the likelihood of success and ensuring that patients have access to cutting-edge therapies. These advancements in clinical trial recruitment and management not only accelerate the pace of research but also improve the prospects for discovering breakthrough cancer treatments [25].

Cost Efficiency and Resource Optimization

Reduced hospital visits: Integrating telemedicine and remote monitoring into digital health brings significant cost-efficiency benefits. These technologies reduce the need for frequent in-person hospital visits, particularly relevant in oncology. Patients can receive consultations and follow-ups from the comfort of their homes, eliminating the need for extensive travel and associated costs. This reduces financial burdens on patients and improves their overall quality of life by minimizing time commitments and discomfort associated with frequent hospital visits. In addition, healthcare providers can optimize their schedules, reducing overhead costs while ensuring timely patient care [26].

Resource allocation: Data analytics, a fundamental component of digital health, empowers healthcare institutions to allocate resources more efficiently. Healthcare providers can gain insights into patient needs, disease trends, and treatment outcomes by analyzing patient data. This information enables them to allocate resources, such as healthcare personnel and equipment, more effectively, ensuring that treatments are delivered where they are needed most. This targeted resource allocation optimizes healthcare delivery, reduces wastage, and lowers costs. It is precious in oncology, where treatment resources are scarce and must be deployed judiciously [27].

Preventative care: Digital health tools excel at identifying patients at high risk of cancer, enabling proactive interventions, and potentially reducing long-term treatment costs. These tools utilize predictive analytics and data from various sources, including genetic, lifestyle, and medical records, to identify individuals with elevated cancer risks. Early interventions, such as lifestyle modifications, regular screenings, or preventive therapies, can be recommended to reduce the likelihood of cancer development. By focusing on preventative care, digital health improves patient outcomes. It helps mitigate the economic burden of treating advanced-stage cancer cases, which are more expensive and resource-intensive [28].

Challenges and ethical considerations

Data Privacy and Security

Data protection: Data privacy and security are paramount in digital health. As digital health systems collect and transmit sensitive patient information, ensuring robust data protection measures is crucial to safeguarding patient privacy. Patient data, which includes personal health information, medical histories, and treatment records, is among the most sensitive and private information individuals possess. Unauthorized access or data breaches can have serious consequences, including identity theft, financial fraud, and using personal health information for malicious purposes. To address these concerns, digital health applications and systems must implement stringent data protection measures, such as encryption, access controls, and secure data storage. These safeguards ensure that patient data remains confidential, accessible only to authorized individuals, and protected against external threats [29].

Cybersecurity risks: The healthcare sector is increasingly vulnerable to cyberattacks, making cybersecurity a pressing concern in digital health. Cybercriminals recognize the value of patient data, making healthcare institutions a prime target for data breaches and ransomware attacks. The consequences of such attacks extend beyond the compromise of patient privacy; they can disrupt healthcare delivery, compromise the integrity of patient records, and even impact patient safety. Ensuring the cybersecurity of digital health platforms and systems is essential to prevent data breaches, maintain the confidentiality and integrity of patient data, and uphold the trust of patients in the security of their health information. Robust cybersecurity measures, including regular system audits, threat monitoring, and employee training, are critical components of a comprehensive security strategy [30].

Regulatory and Compliance Issues

Regulatory framework: The rapidly evolving landscape of digital health presents a significant challenge regarding regulatory and compliance issues. Technological advancements in this field often outpace the development of comprehensive regulatory guidelines. This dynamic environment means healthcare providers and technology developers must navigate complex and continually evolving regulatory landscapes. The challenge lies in ensuring that digital health applications comply with many regulations, including data privacy laws and medical device regulations. This necessitates constant vigilance and adaptability to ensure these technologies meet regulatory requirements while maintaining high patient data protection and safety standards. Striking the right balance between fostering innovation and upholding regulatory compliance is essential for the responsible development and deployment of digital health solutions [31].

Standards and certification: Establishing industry-wide standards and certification processes for digital health applications is critical to improving transparency and safety for patients and healthcare practitioners. The lack of uniform standards in this rapidly expanding field can pose quality control, data security, and interoperability challenges. Standardization efforts seek to define standard criteria that digital health products and solutions must meet to ensure their safety, reliability, and effectiveness. Certification processes, overseen by regulatory bodies or industry organizations, can ensure that digital health applications adhere to these standards. By adhering to established standards and achieving certification, developers can enhance transparency, build trust among users, and ultimately contribute to the responsible and ethical growth of digital health in oncology [32].

Interoperability and Integration

Data fragmentation: One of the pressing challenges in the realm of digital health is the potential for data fragmentation. As digital health systems and devices continue to proliferate, they may need to integrate existing healthcare infrastructure seamlessly. This fragmentation can lead to disparate pockets of health data scattered across various platforms and institutions. Such fragmentation hinders care coordination and collaboration among healthcare providers. It can limit the potential benefits of these technologies by impeding the sharing of critical patient information, potentially resulting in medical errors, delayed treatments, or incomplete medical histories. Addressing data fragmentation is crucial to unlocking the full potential of digital health and providing patients with comprehensive and well-coordinated care [33].

Standardization: Achieving interoperability and standardization in data formats and communication protocols is essential but also challenging. It requires close cooperation among diverse stakeholders, including healthcare providers, technology vendors, and policymakers. Establishing common standards for data exchange and ensuring that digital health systems can communicate effectively is crucial for seamless integration and interoperability. Standardization efforts must encompass various data types, from EHRs to wearable device data and genomic information. It also requires developing and adopting common data exchange protocols and terminology. While standardization can be complex and time-consuming, enabling the efficient flow of information across the healthcare ecosystem is vital to improving care quality, patient safety, and healthcare efficiency [34].

Digital Divide and Access Barriers

Inequality in access: The digital divide poses a significant ethical concern in the context of digital health in oncology. Not all patients have equal access to these transformative technologies. Socioeconomic disparities, geographic location, and varying levels of digital literacy can create a profound digital divide, leaving some individuals without the essential benefits of digital health tools. Patients from economically disadvantaged backgrounds or rural areas may need help accessing the internet, smartphones, or other necessary devices, hindering their ability to participate in telemedicine, remote monitoring, or mobile health applications. This inequality in access threatens to exacerbate health disparities, as those who need advanced healthcare solutions the most may be the least able to access them [35].

Healthcare disparities: Adopting digital health in oncology can exacerbate healthcare disparities without proper attention and intervention. Vulnerable populations, such as racial and ethnic minorities, individuals with lower socioeconomic status, and those with limited access to healthcare facilities, may be disproportionately affected. Failure to address these disparities could result in unequal access to early cancer detection, personalized treatments, and patient support services. Healthcare systems and policymakers must prioritize efforts to bridge these gaps, ensuring that digital health tools reach and benefit all population segments equitably [36].

Ethical Use of Patient Data

Informed consent: Ethical considerations regarding the use of patient data in digital health applications begin with the critical issue of informed consent. Obtaining informed consent is a multifaceted challenge in this context. Patients must be provided with clear and comprehensible information about how their data will be collected, processed, and used. Consent mechanisms should be transparent and easily accessible, ensuring patients can make informed choices about using their health information. This includes understanding the potential risks, benefits, and limitations of data sharing in digital health. Striking a balance between respecting individual autonomy and facilitating medical advancements is paramount to ensuring ethical data use [37].

Data ownership: The ethical landscape surrounding data ownership and control in digital health is complex. Patients have a rightful interest in how their health data is used, but defining the boundaries of data ownership can be challenging. While individuals should have a say in using their data, acknowledging the collective value of health data for medical research and advancements is equally essential. Striking the right balance between patient autonomy and the greater good of advancing medical knowledge is a delicate ethical consideration. Establishing clear policies and frameworks that respect individual rights while promoting the responsible sharing and utilization of data is crucial [38].

Data de-identification: Ensuring the anonymity of patient data when sharing it for research purposes is a critical ethical imperative. While data sharing is essential for scientific progress, preserving patient privacy is equally essential. De-identifying patient data-removing personally identifiable information while retaining its utility for research-poses ethical challenges. Striking the right balance between protecting individual privacy and facilitating research is essential. Ensuring robust de-identification methods, data security measures, and strict adherence to ethical guidelines will be paramount in addressing these challenges [39].

Future trends and directions

Advancements in AI and Machine Learning

AI-driven precision medicine: The ever-advancing field of AI and machine learning is poised to reshape oncology through precision medicine. As these technologies evolve, they will enable healthcare providers to make more accurate and personalized predictions regarding cancer risk. By harnessing the power of AI-driven algorithms, healthcare professionals can analyze vast datasets, including genetic information, lifestyle factors, and medical histories, to identify individuals at the highest risk of developing cancer. Moreover, AI will enable the generation of highly personalized treatment recommendations based on a patient's unique genetic and clinical profile. This tailoring of treatments ensures that patients receive more effective therapies associated with fewer adverse effects. Furthermore, AI will empower real-time treatment adjustments based on patient responses and emerging research findings, ushering in an era of dynamic and adaptable cancer care [40].

Drug discovery: AI is set to play a pivotal role in revolutionizing the drug discovery process in oncology. AI-driven algorithms will excel in identifying novel therapeutic targets, predicting drug interactions, and optimizing the development of targeted therapies and immunotherapies. These algorithms can analyze vast datasets of biological and chemical information to identify potential drug candidates, predict their efficacy, and even anticipate potential side effects or drug-drug interactions. By accelerating the drug discovery pipeline, AI holds the potential to significantly reduce the time and costs associated with bringing new cancer treatments to market. This accelerated drug discovery benefits patients by providing them with more treatment options and contributing to our overall understanding of cancer biology, opening doors to innovative and precisely targeted therapies [41].

Integration of Virtual Reality and Augmented Reality

Immersive training and education: Integrating virtual reality (VR) and augmented reality (AR) into digital health in oncology promises to revolutionize medical education and training. These immersive technologies offer healthcare professionals a dynamic and risk-free environment to practice and refine their surgical procedures and treatment protocols. Surgeons can perform intricate surgical simulations, oncologists can rehearse complex treatment procedures, and radiologists can practice interpreting medical images in a highly realistic yet controlled virtual setting. This immersive training not only enhances the skill set of healthcare providers but also reduces the potential risks associated with real-life learning, ultimately leading to improved patient outcomes and safety [42].

Patient engagement: Beyond professional training, VR and AR technologies are set to enhance patient engagement and education in oncology. By leveraging these technologies, healthcare providers can offer patients interactive, 3D visualizations of their conditions and treatment options. Patients can explore virtual representations of their tumors, view treatment procedures in detail, and gain a deeper understanding of their diagnoses-all in a highly accessible and comprehensible format. This immersive patient education approach promotes informed decision-making and empowers individuals to actively participate in their care journeys. It reduces anxiety, fosters a sense of control, and bridges the gap between complex medical information and patient comprehension, ultimately leading to improved treatment adherence and patient satisfaction [43].

Predictive Analytics and Early Intervention

Early detection: The future of digital health in oncology promises increasingly sophisticated predictive analytics tools that will revolutionize early cancer detection. These advancements will be achieved by integrating and analyzing diverse data sources, including genetic information, lifestyle factors, and medical imaging data. By applying cutting-edge algorithms to this wealth of information, predictive analytics will empower healthcare providers to identify the earliest signs of cancer development with unprecedented accuracy. This capability is not limited to a single data type but encompasses a holistic approach that considers multifaceted aspects of a patient's health. As a result, cancers can be identified at their nascent stages, often before symptoms manifest, offering the potential for earlier, more effective treatment strategies and improved patient outcomes [44].

Proactive care: Predictive analytics, coupled with digital health technologies, will enable healthcare providers to transition from a reactive approach to a proactive one in cancer care. These tools will empower clinicians to identify high-risk individuals, often long before cancer develops. By leveraging predictive models and comprehensive patient data, healthcare providers can personalize interventions and monitoring for those at elevated risk of developing cancer. This proactive approach may involve lifestyle modifications, tailored screenings, or preventive therapies, all designed to mitigate risk factors and intervene before cancer can develop or progress. Ultimately, this shift toward proactive care holds the potential to significantly reduce the incidence and impact of cancer, representing a pivotal advancement in oncology [45].

Global Collaboration and Data Sharing

Data pools: In the ever-evolving landscape of digital health in oncology, international collaboration and data-sharing initiatives are cornerstones of progress. These endeavors unite healthcare institutions, researchers, and data scientists worldwide to create vast pools of cancer-related data. These expansive datasets encompass diverse patient profiles, tumor types, treatment outcomes, and more. By pooling this wealth of information, scientists and researchers gain unprecedented access to a global knowledge repository. This collective effort facilitates large-scale research efforts, transcending geographic boundaries and enabling the analysis of cancer trends and patterns on a previously unimaginable scale. These data pools become invaluable treasure troves, offering insights into cancer biology, treatment efficacy, and patient outcomes that transcend regional variations [28].

Treatment standardization: Global collaboration and data sharing advance our understanding of cancer and contribute significantly to standardizing cancer treatment protocols. Evidence-based best practices and guidelines emerge as data from diverse populations and healthcare systems is shared and analyzed. This standardization ensures that patients worldwide receive the most effective and evidence-based care, regardless of location. It helps eliminate geographical disparities in cancer treatment by providing healthcare professionals with a comprehensive understanding of what works best in different contexts. Treatment decisions become increasingly informed by a global knowledge base, leading to improved patient outcomes, reduced variations in care, and a more equitable distribution of the benefits of cutting-edge treatments [46].

Policy and Regulatory Changes

Adaptive regulation: In response to the rapid evolution of digital health technologies, regulatory bodies worldwide recognize the need for more flexible and adaptive regulatory frameworks. These frameworks are designed to keep pace with innovation and encourage it while maintaining a steadfast commitment to patient safety and data privacy. The traditional regulatory pathways often need to be better suited to the dynamic nature of digital health, which encompasses a wide range of technologies, from wearable devices to AI-powered diagnostics. Adaptive regulation seeks to balance fostering innovation and ensuring that these innovations meet rigorous safety and privacy standards. It promotes a more agile and responsive approach to regulatory oversight, allowing novel technologies to reach patients more quickly while minimizing risks [32].

Interoperability mandates: Policymakers and healthcare authorities increasingly emphasize the importance of interoperability and data exchange among digital health systems. Interoperability mandates aim to create a more seamless and connected healthcare ecosystem where patient information can be securely accessed and shared among healthcare providers, institutions, and technologies. This push for greater interoperability is driven by the recognition that siloed health data can impede care coordination and limit the potential benefits of digital health. By standardizing data formats, communication protocols, and data-sharing practices, these mandates enable patients and healthcare providers to access and exchange health information efficiently and securely, ultimately leading to more integrated and patient-centered care [47].

## Conclusions

The landscape of digital health in oncology is marked by incredible promise and transformative potential. As we have explored its diverse applications, benefits, challenges, and ethical considerations, it becomes evident that digital health is reshaping how we approach cancer care and research. The personalized diagnostics, treatment recommendations, and early interventions it offers hold the potential to revolutionize patient outcomes and experiences. However, as we step into this future, we must remain vigilant in addressing ethical concerns, ensuring data privacy, and advocating for equitable access. Collaboration among stakeholders, from healthcare providers to policymakers and technology developers, is paramount to realizing the full potential of digital health. It is a call to action that places the patient at the center of care and promises a future where the burden of cancer is lightened through innovation and compassion.
